# Experience-dependent plasticity in early stations of sensory processing in mature brains: effects of environmental enrichment on dendrite measures in trigeminal nuclei

**DOI:** 10.1007/s00429-021-02424-3

**Published:** 2021-11-22

**Authors:** Yasmina B. Martin, Pilar Negredo, Carlos Avendaño

**Affiliations:** 1 Departamento de Anatomía, Facultad de Medicina, Universidad Francisco de Vitoria, Pozuelo de Alarcón, Madrid Spain; 2 Department of Anatomy, Histology and Neuroscience, Medical School, Autónoma University of Madrid, Madrid, Spain

**Keywords:** Dendritic trees, Barrels, Enriched environment, Morphometry, Adult brain plasticity, Dextran amine

## Abstract

Nervous systems respond with structural changes to environmental changes even in adulthood. In recent years, experience-dependent structural plasticity was shown not to be restricted to the cerebral cortex, as it also occurs at subcortical and even peripheral levels. We have previously shown that two populations of trigeminal nuclei neurons, trigeminothalamic *barrelette* neurons of the principal nucleus (Pr5), and *intersubnuclear* neurons in the caudal division of the spinal trigeminal nucleus (Sp5C) that project to Pr5 underwent morphometric and topological changes in their dendritic trees after a prolonged total or partial loss of afferent input from the vibrissae. Here we examined whether and what structural alterations could be elicited in the dendritic trees of the same cell populations in young adult rats after being exposed for 2 months to an enriched environment (EE), and how these changes evolved when animals were returned to standard housing for an additional 2 months. Neurons were retrogradely labeled with BDA delivered to, respectively, the ventral posteromedial thalamic nucleus or Pr5. Fully labeled cells were digitally reconstructed with Neurolucida and analyzed with NeuroExplorer. EE gave rise to increases in dendritic length, number of trees and branching nodes, spatial expansion of the trees, and dendritic spines, which were less pronounced in Sp5C than in Pr5 and differed between sides. In Pr5, these parameters returned, but only partially, to control values after EE withdrawal. These results underscore a ubiquity of experience-dependent changes that should not be overlooked when interpreting neuroplasticity and developing plasticity-based therapeutic strategies.

**Supplementary Information:**

The online version contains supplementary material available at 10.1007/s00429-021-02424-3.

## Introduction

Sustained alterations in sensory experience induce structural changes in the brain not only during development and at early maturational stages, but also throughout life. This property started to be explored soon after Hebb formulated his influential hypothesis that lasting structural changes, consisting of increases in number and/or size of interneuronal contacts, should occur whenever a cell repeatedly excites another cell (Cooper 2005; Hebb 1949). For many years such phenomena were assumed to be largely circumscribed to the cortex, where plastic modifications of synapses, spines, axons, and/or somatodendritic domains would take place in response to sensory deprivation or overstimulation (Feldman 2009; Feldman and Brecht 2005; Hickmott and Steen 2005; Holtmaat and Svoboda 2009; Knott et al. 2002; Ma et al. 2016; Machin et al. 2006; Schubert et al. 2013; Tailby et al. 2005). These changes, in turn, were proposed to underlie learning (Geinisman et al. 1993; Rusakov et al. 1997) and functional reorganization after injury (Florence et al. 1998; Garraghty et al. 2006; Keck et al. 2011; Mowery et al. 2015; Schubert et al. 2013). Experience-dependent structural plasticity in lower sensory centers was seldom tackled, however, in spite of the early demonstration of intraspinal sprouting of primary afferents after partial local deafferentation (Liu and Chambers 1958), and growing data on the functional reorganization in sensory domains of the spinal cord and brain stem after sensory input manipulations (Churchill et al. 2001; Devor and Wall 1978, 1981; Panetsos et al. 1995; Pettit and Schwark 1993). More recently, we described lasting changes in the morphometry and topology of dendrites of second-order sensory neurons and terminal arbors of primary sensory afferents not only after peripheral nerve injury, but also after sustained removal of haptic touch by unilateral whisker trimming (Fernández-Montoya et al. 2018; Martin et al. 2014; Negredo et al. 2009).

Environmental enrichment (EE) is a widely used strategy to increase the exposure of experimental subjects to sensory stimulation and promote voluntary social interactions and exploratory behaviors (rev. in Alwis and Rajan 2014; Ball et al. 2019; Diamond 1988; Mohammed et al. 2002; van Praag et al. 2000). EE gives rise to a range of structural, molecular and functional effects in the brain, which have been proposed to underlie improvements in learning and cognitive abilities, and promote functional improvements in developmental disorders or recovery after lesions in adults (Nithianantharajah and Hannan 2006). As for other paradigms of experience-dependent plasticity, studies of the EE effects have been essentially restricted to cortical structures. Such effects include gains in cortical weight, thickness, volume and acetylcholinesterase activity (Diamond et al. 1976; Machín et al. 2004; Rosenzweig et al. 1962), increased dendritic branching and length (Greenough et al. 1973; Hickmott and Ethell 2006; Uylings et al. 1978; Volkmar and Greenough 1972), synaptogenesis and addition of new spines (Globus et al. 1973; Johansson and Belichenko 2002; Jung and Herms 2014; Landers et al. 2011), expansion in number and volume of small blood vessels (Black et al. 1991; Ekstrand et al. 2008; He et al. 2017), increased volume and fiber length of subcortical white matter (Yang et al. 2015), glial plasticity (Ehninger and Kempermann 2003; Keiner et al. 2017; Sirevaag and Greenough 1991; Viola et al. 2009), and lasting and dynamic changes in genes encoding a variety of transcription factors (Valles et al. 2011).

The cerebral cortex is certainly where more prominent experience-dependent changes take place following exposure to EE. However, we have recently shown significant changes at molecular and cellular levels in sensory ganglia and primary afferents from sensory neurons in young adult rats after 7–8 weeks of exposure to EE. The trigeminal ganglion on the right side displayed extensive down-regulation of a number of glutamate transmission-related genes, without alterations at the protein level, which increased the left–right asymmetry seen in naïve animals and suggested an enhancement of glutamatergic transmission (Fernandez-Montoya et al. 2016). Moreover, EE induced significant and widespread increments in the trigeminal nuclei in the number and size of terminal and *en passant* varicosities of putatively myelinated afferents (Fernández-Montoya et al. 2018).

The present study aims at examining the effects that prolonged exposure to EE may cause on the dendritic arbors of two populations of second-order trigeminal neurons, both of which are targets for primary afferents from sensory neurons in the trigeminal ganglion (TG). One population consists of the *barrelette* neurons of the principal trigeminal nucleus (Pr5), which are the main source of the lemniscal pathway to the thalamus (Urbain and Deschênes 2007); the other corresponds to intersubnuclear neurons in the rostral part of the caudal division of the spinal trigeminal nucleus (Sp5C), which synapse on barrelette and other thalamic-projecting neurons conveying information from the vibrissae, shaping their receptive fields (Jacquin et al. 1990; Martin et al. 2014). Moreover, it will be tested whether the changes found in the barrelette neurons after a period of EE are transitory and reversible by returning animals to a standard housing for the same period before performing the terminal tracing experiments.

## Materials and methods

### Subjects and experimental procedures

Young adult male Sprague–Dawley rats (*n* = 44) from our own colony, originating from Harlan (Harlan Iberica, Barcelona, Spain) were used. All animal procedures were approved in advance by the Ethics in Research Committee of the Autónoma University of Madrid, in accordance with European Community’s Council Directive 2010/63/UE. Every effort was made to minimize the suffering of the animals, as well as the number of animals used. After weaning, at P21, rats were tagged and housed under standard colony conditions [4 rats/cage (28 × 48 × 28 cm)] and maintained on a reversed 12:12-h dark/light cycle with ad libitum access to food and water. The animals were divided into three groups: Control group (C; *n* = 23), rats subjected to minimal handling for routine cleaning; enriched environment group (E, *n* = 16), rats daily exposed to an EE for 2 months; and Enrichment Withdrawal group (EW; *n* = 5), animals that after 2 months of EE were returned for an additional 2 months to standard housing. For enrichment, animals that reached the age at which they are considered young adults (between 63 and 70 days old) were placed 3 h per day, 5 days a week during the activity cycle (dark conditions), in groups of eight animals in an enrichment and socialization methacrylate cage (80 × 100 × 60 cm) with a metal wire mesh cover. This cage had platforms and ramps at different heights, and contained various toys with different textures: tubes, boxes, platforms and ramps as "non-natural" elements, and pinecones, stones, sticks and bowls with water as “natural” elements. Every day, rewards (sweetened puffed wheat cereal) were placed in different places in the cage and the position of platforms and ramps as well as toys are changed twice a week. Running wheels were not included, to avoid the well-known interfering factors that wheel running introduces in rats’ energy balance and behavior (Novak et al. 2012). The handling of the animals during this period was carried out with emission light inside the red (700 nm) that allowed the experimenters to see inside the room but did not interfere with the activity of the rats since they are blind to that color. Animals did not show behavioral signs of stress, and their body weight, which was controlled at different times of the experiment, showed no differences between groups.

### Retrograde labeling

At the end of the stipulated survival time depending on each group (2 months E and C, 4 months EW), rats were anesthetized with ketamine (55 mg/kg)–xylazine (15 mg/kg)–atropine (0.2 mg/kg) and their heads were positioned in a stereotaxic frame. Bilateral microiontophoretic injections of biotinylated dextran amine (BDA 3000, Invitrogen-Molecular Probes, Eugene, OR, USA) were made with a Midgard constant current source (Stoelting, Wood Dale, IL; 7 μA positive current, 7-s on–off cycle, 6–10 min). Injections were guided by the presence of short-latency, high-amplitude single- and multiunit responses to gentle mechanical stimulation of the vibrissal pad, recorded through the glass micropipettes (A-M Systems, Sequim, WA; 10–15 μm tip outer diameter). The targets were the ventral posteromedial nucleus of the thalamus (VPM) and the principal trigeminal nucleus (Pr5), to retrogradely label Pr5 trigeminothalamic barrelette neurons and trigeminal intersubnuclear neurons in the caudal division of the spinal trigeminal nucleus that project to Pr5 (Sp5C-Pr5), respectively (Martin et al. 2014; Negredo et al. 2009).

Ten days later, the rats were deeply anesthetized (Dolethal, 50 mg/kg i.p.) and perfused through the ascending aorta with 0.9% NaCl (100 ml, 2 min), followed by 4% paraformaldehyde in 0.1 M phosphate buffer (PB; pH 7.4, 1000 ml, 20 min, 10–12 ºC). The brains were removed and blocked depending on whether injections were made in the thalamus or in Pr5. In the first case two blocks were obtained, one containing the thalamic region that received the tracer deposits and the other containing the whole rostrocaudal extent of Pr5. In cases injected in Pr5 only one block was prepared that included the whole rostrocaudal extent of the brainstem, from the rostral tip of Pr5 to segment C1 of the spinal cord. All blocks were postfixed in the same fixative overnight at 4 ºC, subsequently cryoprotected for 2 days in 30% sucrose in 0.1 M PB, and then frozen and serially cut at 50 µm in the coronal plane in a sliding microtome. For precisely assessing the injection location and extent, one-third of the sections of the thalamus and one-third of the sections of Pr5 which included the area of injection, were incubated in avidin–biotin peroxidase (Kit ABC EliteVR, 1:250 in PBS; Vector, Burlingame, CA) and revealed with diaminobenzidine (DAB, 0.05% in PBS; Sigma, St. Louis, MO) with H_2_O_2_ added (0.003% of the stock 30% solution). Alternate thalamic and Pr5 series were Nissl-stained with 0.1% Cresyl Violet, or reacted for cytochrome oxidase (Wong-Riley 1979). For recovering the retrogradely labeled neurons in Pr5 or Sp5C, all the corresponding brainstem sections were serially collected and similarly revealed with ABC-DAB.

### Neuron selection and dendrite morphometry

**Fig. 1:**
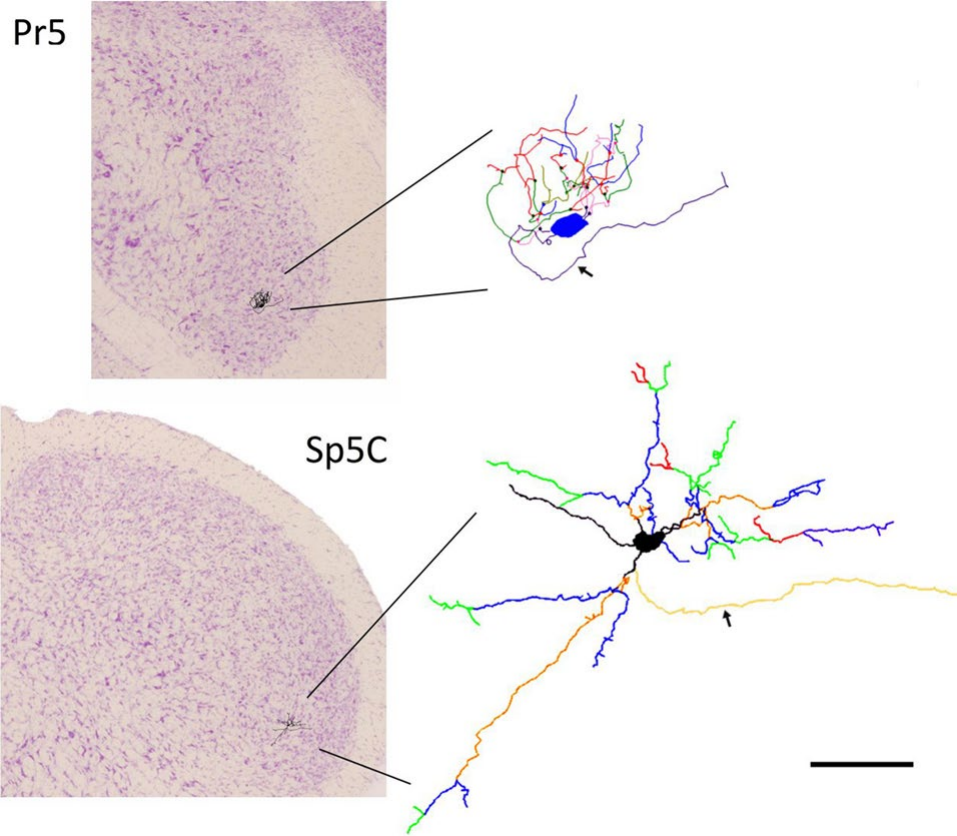
Illustration of two Nissl-stained coronal sections through the Pr5 (top) and Sp5C (bottom) trigeminal nuclei on which line drawings of actual labeled neurons are shown at the approximate size and locations where they appeared. The digital reconstructions of these neurons with Neurolucida are shown at greater magnification on the right. Axons are indicated with small arrows. Scale bar 100 μm (applicable to the reconstructed neurons)

BDA retrogradely labeled neurons were selected with strict and consistent criteria, and their dendritic trees were digitally reconstructed and analyzed after correcting for shrinkage in the *z*-axis, using Neurolucida system with a 40 × dry objective (Zeiss *N*-Achroplan, NA 5 0.65) and NeuroExplorer software (MicroBrightField, Williston, VT, USA; Fig. 1). As in our previous studies (Martin et al. 2014; Negredo et al. 2009) neurons had to fulfill three requirements to be included in the study. Firstly, they had to be located in the appropriate region for each neuron population: the barrelette region of Pr5, or the rostroventral sector of the Sp5C, where most were located in lamina III and somewhat fewer in laminae II and IV. Secondly, neurons had to display apparently complete labeling of their soma and dendritic trees, judging by the presence of well-defined dendritic ends within the tissue sections, and they could be clearly separated from other neighboring neurons, to avoid ambiguities in tracking separate dendrites. In most cases, only three to six neurons fulfilled these criteria, and were reconstructed on each side. When the population of satisfactory candidate neurons was larger, only up to six neurons were selected by systematic random sampling throughout the nucleus. The authors who executed the digital reconstructions (YBM and PN, with occasional crosschecks between the two by random repetition of some neurons) were blinded to the brain side and subject’s group.

From the vectorized trees (by tree denoting each dendritic arbor arising from a single, primary dendritic trunk) we prepared dendrograms that included measures of node number (node: branching point of a dendrite), tree degree (number of terminal segments of a tree), segment order (segment; portion of a dendritic shaft between two nodes—intermediate segment*-*, or between the soma and the first node, or between the last node and the dendritic end—terminal segment-; order*:* centrifugal position of the segment from the soma, assigning order 1 to primary dendrites before their first division), path length, and, only for Sp5C, spine number. Because of their low numbers, spines were not reconstructed in Pr5. Also, we performed 3D Sholl analyses (Sholl 1953) in 20 µm (Pr5 neurons) or 30 µm (Sp5C neurons) increments, which included measures of radial Euclidean distance for node numbers, segment length densities, segment intersections, spine number, etc. Data were computed both for single trees and for whole neurons. Data analysis and statistics were performed only when all the reconstructions had been completed.

### Statistical analyses

Statistical testing was performed using Statgraphics plus (ver. 4.0 for Windows) and SPSS (ver.15.0 for Windows). Point graphs were generated with Graphpad Prism ver. 5.03 (GraphPad Software Inc., San Diego, CA, USA). For graphic rendering of Sholl data, curves and pointwise bootstrap confidence intervals were generated with Real Statistics Resource Pack software (rel 7.6; Zaiontz 2020), and two-dimensional density (heat) maps were created with SigmaPlot 8.0. Comparisons between sides within the same group, between experimental groups, or between each experimental group and the control group were made using single neurons across each side and group as sample units. Intergroup statistical comparisons for each relevant parameter were made taking two groups at a time. Comparisons of single parameters between sides and groups were made using unpaired parametric (Student’s *t* test) or nonparametric tests (Mann–Whitney), after testing for normality and homogeneity of variances. To assess differences in the distribution of morphometric events at selected distances from the soma in Sholl analyses, two-way analysis of variance (ANOVA) with post hoc tests were used, and the Mann–Whitney test was applied pointwise to bootstrapped distributions. The distributions of dendritic trees according to their maximum Euclidean distance were plotted in Excel, and were compared between groups using the two-sample Kolmogorov–Smirnov *D* test (Kirkman 1996). In graphs, differences are reported as significant (*0.01 < *p* < 0.05), and very significant (**p < 0.01); for other symbols, see legends.

## Results

### The Pr5-thalamic *barrelette* and the intersubnuclear Sp5C-Pr5 neurons: retrograde labeling and general morphology

The location of BDA injections and the retrogradely labeled neurons in control animals were described in detail in our previous studies (Negredo et al. 2009; Martin et al. 2014), and the new cases added here were consistent with those descriptions (Tables 1, 2). Briefly, barrelette neurons in Pr5 projecting to the contralateral VPM have small bodies from which 3–5 primary dendrites emerge that branch variably, predominating low-complexity (degrees 1–4) over more complex trees (degree > 5) in a 2:1 proportion. Dendrites vary in thickness, display frequent swellings and are largely spineless. Most dendritic trees are highly polarized, probably depending on their location within the thalamic *barreloid* (undetectable in our material), as shown by digital 3D reconstructions and polar histograms. Intersubnuclear neurons in Sp5C projecting ipsilaterally to Pr5 have multipolar ovoid or fusiform bodies that appear more often, but not exclusively in lamina III. From 4–5 (range, 2–8) primary dendrites emerge dendritic arbors that ramify mainly in the same lamina as the cell body. These arbors lack any definite polarization, and vary in size, occasionally reaching high complexity (up to degree 25), but in most cases being restricted to degrees 1–12.

**Table 1:** Global morphometric and topologic data (mean ± SEM) of the Pr5 *barrelette* neurons

Group^**§**^	Rats (*n*)	Hemispheres (*n*)	Neurons (*n*)	Primary dendrites (*n*)	Nodes (*n*)	Terminal segments (*n*)	Total dendritic length (μm)
C-L	13	10	35	4.60 ± 0.21^†^	18.11 ± 1.53	22.77 ± 1.49	1237 ± 102^†^
C-R		7	28	3.75 ± 0.25	14.64 ± 0.98	18.36 ± 1.00	780 ± 49
E-L	6	4	16	4.19 ± 0.37	24.75 ± 2.29*	29.25 ± 2.50*	1114 ± 116
E-R		5	14	4.86 ± 0.36*	20.43 ± 2.22*	25.43 ± 2.23**	1169 ± 95**
EW-L	5	4	12	4.00 ± 0.35	20.17 ± 3.00	24.17 ± 3.07	1538 ± 224
EW-R		4	12	4.00 ± 0.33	17.67 ± 3.01	21.67 ± 3.05	1137 ± 177*

*Greater than the corresponding value on the same side in Controls (two-tailed: *p < 0.05; **p < 0.01).

^**§**^L and R stand for left and right sides, respectively

^†^Significantly more primary dendrites (*p* = 0.006; two-tailed) and greater dendritic length (*p* < 0.001; two-tailed) on the left than on the right side

**Table 2:** Global morphometric and topologic data (mean ± SEM) of the Sp5C neurons

Group^**§**^	Rats (*n*)	Hemispheres (*n*)	Neurons (*n*)	Primary dendrites (*n*)	Nodes (*n*)	Terminal segments (*n*)	Spines (*n*)	Total dendritic length (μm)
C-L	10	7	35	4.26 ± 0.15	27.1 ± 1.7	31.4 ± 1.7	19.7 ± 4.4	2657 ± 302
C-R		7	33	4.30 ± 0.19	26.3 ± 2.6	30.6 ± 2.7	19.2 ± 4.4	2285 ± 205
E-L	11	6	30	3.88 ± 0.20	28.8 ± 1.2	32.6 ± 1.3	29.8 ± 11.5	3076 ± 362
E-R		6	28	4.35 ± 0.28	31.7 ± 3.1	36.0 ± 3.2	24.1 ± 3.6	3134 ± 292*

*Greater total dendritic length, compared with the same side of Controls (*p* = 0.033; two-tailed)

^**§**^L and R stand for left and right sides, respectively.

Dendritic arbors differed between sides. Pr5 barrelette neurons exhibited a significant interhemispheric asymmetry in their dendritic trees in control conditions (Negredo et al. 2009), having 23% more primary dendrites on the left, where total dendritic length per neuron was 59% greater than on the right (Table 1). The increased length resulted from more numerous simplest (order one) arbors, and longer dendritic segments in general, particularly so the terminal segments of complex trees, and was associated to greater tortuosity and longer maximum Euclidean extension (Negredo et al. 2009). Intersubnuclear Sp5C neurons, in contrast, showed only a non-significant 16% greater dendritic length on the left (Table 2), mainly attributable to longer expanse of terminal segments.

### Effects of long-term repeated exposure to an enriched environment

Enrichment induced a pronounced increase in all global parameters on the right side, and in the number of nodes and terminal segments (but not dendritic length) in the left Pr5 barrelette neurons, compared to the corresponding sides in controls (Table 1). One collateral effect of these changes was the disappearance of side asymmetries. Dendritic trees in intersubnuclear Sp5C neurons after enrichment showed moderate gains in these parameters on the right, which reached statistical significance only in total dendritic length (Table 2).

**Fig. 2:**
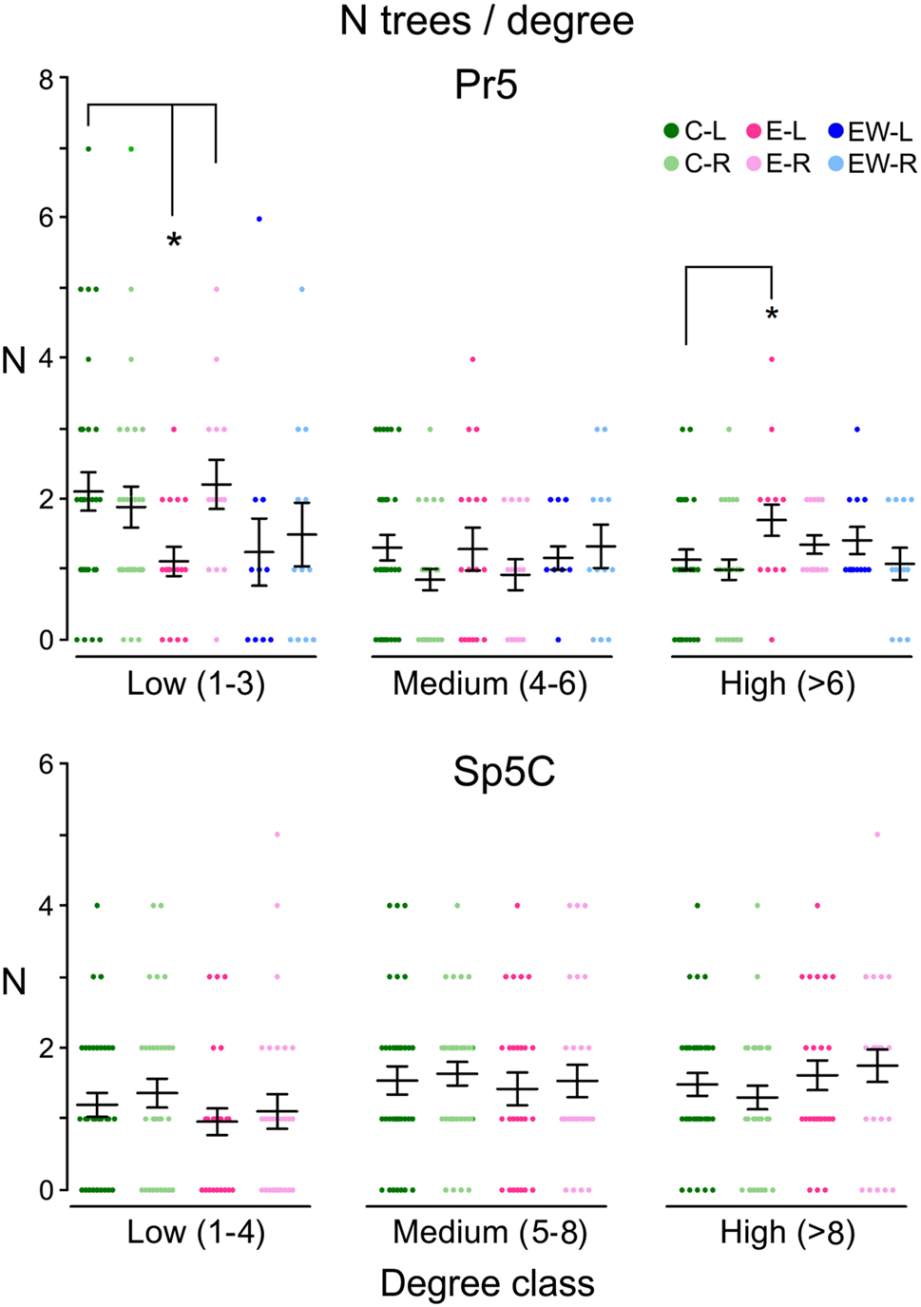
Relative presence of dendritic trees classed by degree as of low (degree 1–3 for Pr5; 1–4 for Sp5C), medium (4–6 for Pr5; 5–8 for Sp5C) and high (> 6 for Pr5; > 8 for Sp5C) branching complexity. The scatter plots display individual data points with bars showing means ± SEM. Number of trees for Pr5: 160 (C-L), 105 (C-R), 64 (E-L), 63 (E-R), 46 (EW-L) and 47 (EW-R). Dark- and light-colored bars represent the left and right sides, respectively. In Pr5, environmental enrichment produced a relative decrease of less complex trees on the left side (*p* = 0.027, two-tailed *T* test), counterbalanced by a proportional increase of the most complex ones (*p* = 0.045). This significant gain in complexity in Pr5 neurons on the left side disappeared after enrichment withdrawal. Neurons on the right Pr5 or on either side of Sp5C did not differ from controls

A more detailed analysis of the trees showed that the increase in primary dendrites on the right Pr5 occurred without altering the distribution of trees according to their branching complexity. This did not happen on the left side, where a significant decrease of lower degree trees was counterbalanced by an increase of more complex trees, with no changes in the mean number of primary dendrites (Fig. 2). Sp5C neurons did not display differences between groups.

**Fig. 3:**
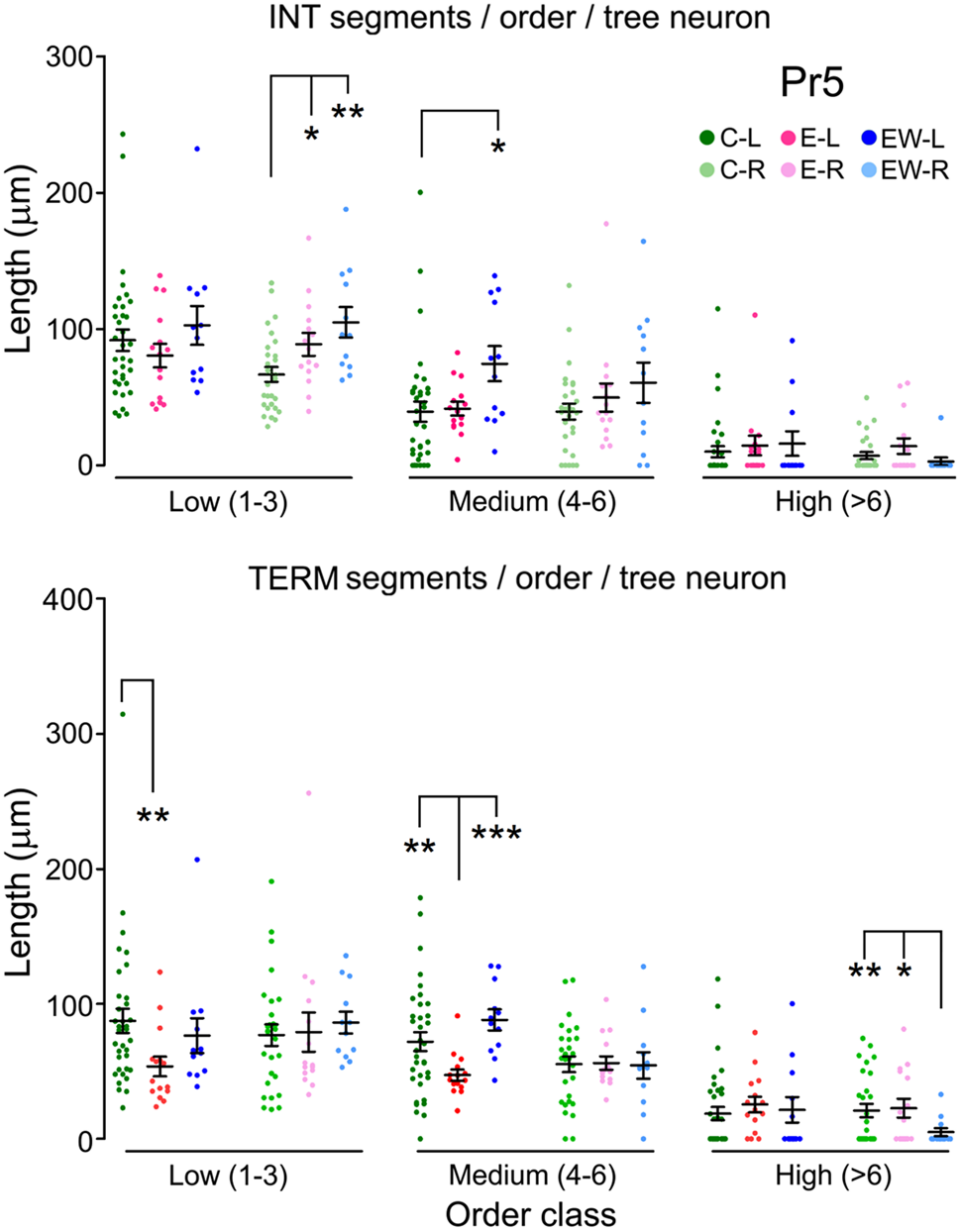
Contribution to total dendritic length of intermediate (top) and terminal (bottom) dendritic segments grouped as of low (orders 1–3), medium (4–6) or higher order (> 6) in barrelette neurons of Pr5. The scatter plots display individual data points of the summed lengths for each order class per tree and neuron (*N* between 12 and 35, see Table 1). Bars represent means ± SEM. Color codes as in Fig. 2. Between sides differences were noticed in intermediate segments, which were significantly longer on the left in group C, low order class (*p* = 0.011, not shown), and terminal segments in group EW, medium order class (*p* = 0.013, not shown). Comparisons between groups of intermediate segments, showed no differences on the left in low- and high-order segments, but medium-order segments were longer in group EW. On the right, controls showed shorter segments of low and medium order compared with groups E and EW, but this difference reached significance only for low-order segments. Terminal segments had similar mean length in C and EW groups on the left, while those in the E group were significantly shorter in low- and medium-order segments. On the right, there were no differences between groups, except for a significant decrease in length of the high-order segments in the EW group. Two-tailed comparisons. *p < 0.05; **p < 0.01; ***p < 0.001

**Fig. 4:**
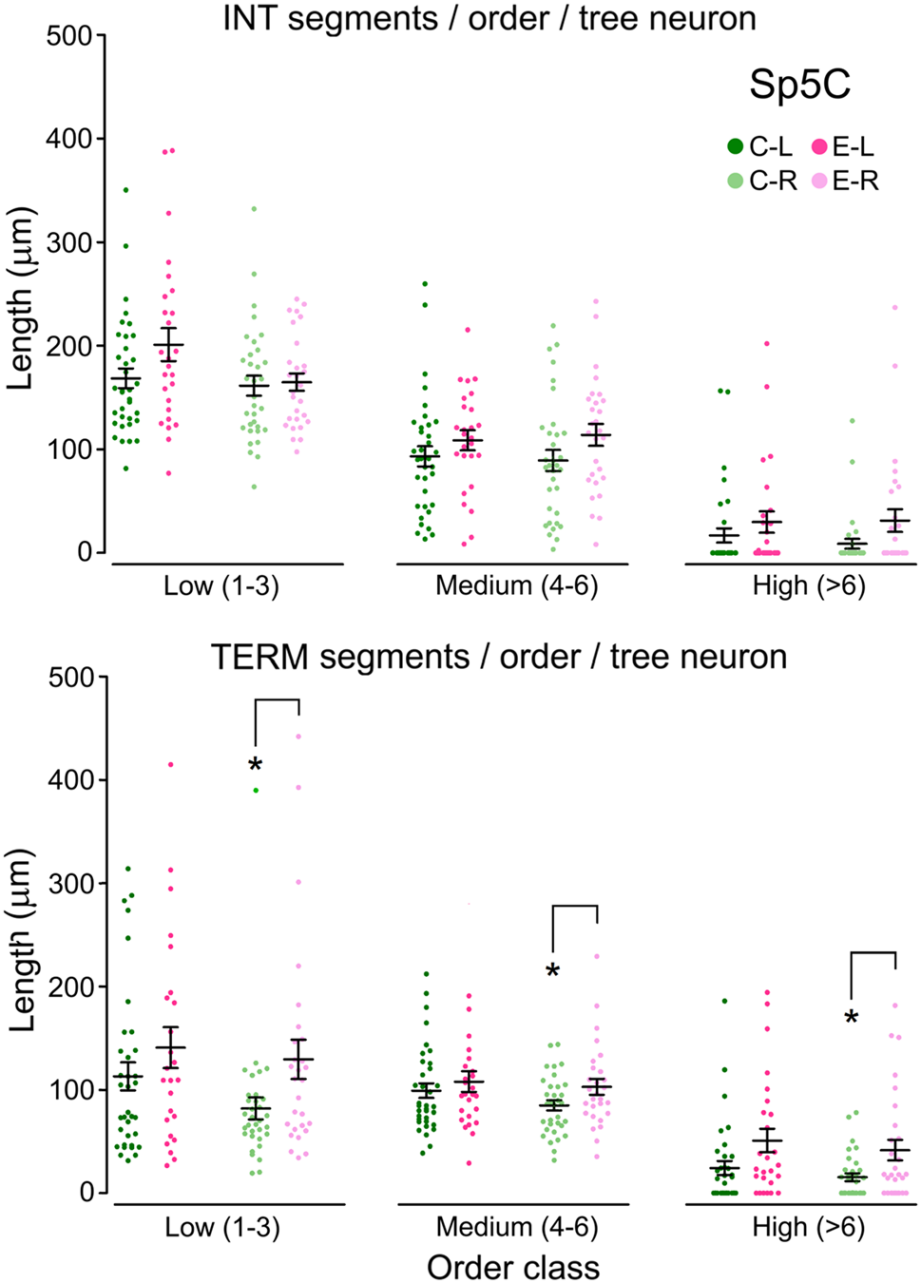
Contribution to total dendritic length of intermediate (top) and terminal (bottom) dendritic segments grouped as low (orders 1–3), medium (4–6) or higher order (> 6) in intersubnuclear Sp5C neurons. Groups, codes and symbols as in Fig. 3 (*N* between 28 and 35, see Table 2). Neither intermediate nor terminal segments showed differences between sides in any group. Compared to controls, enrichment did not produce significant changes in segment length in any order class on intermediate segments. In terminal segments, however, there was a global increase in length in segments of all orders, which reached significance on the right side. Two-tailed comparisons. *p < 0.05

**Fig. 5:**
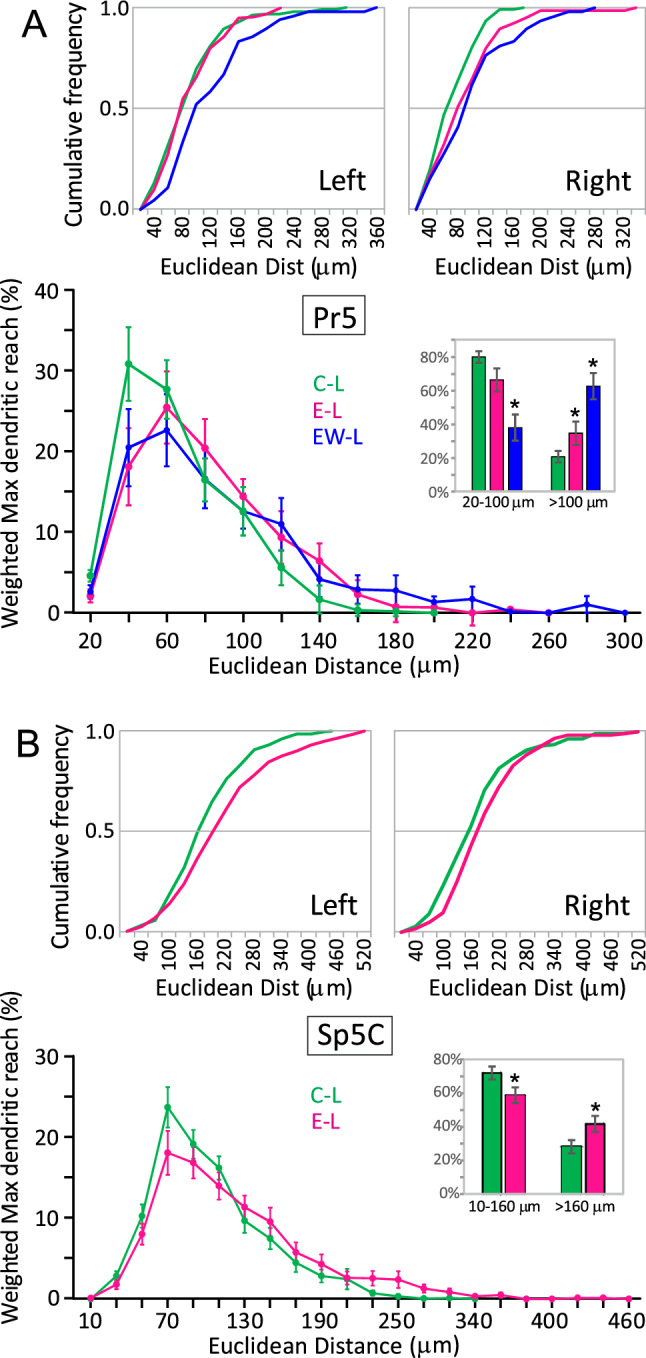
Panels showing the maximum reach of dendritic trees of Pr5 (A) and Sp5C (B) neurons. Each panel presents the cumulative distributions of maximum Euclidean distance for each group, using individual trees as sampling units (top), and the relative contribution of the mean maximum distance reached, using whole neurons as sampling units and weighting Euclidean distances across trees for each neuron (bottom). These distances were also pooled into short-range and long-range categories for further analysis (bar histograms). **A** On the left side, the cumulative distribution of maximum tree lengths did not change after enrichment compared to controls, but increased significantly after enrichment withdrawal (*p* = 0.013; two-tailed Kolmogorov–Smirnov *D* test). This effect in the EW group was accompanied by a significant increase in neurons with greater average dendritic reach and a parallel decrease in neurons with shorter trees (*p* < 0.05, mean ± sem; two-tailed Student’s t test). A similar pattern was found in the E group, but the difference with controls reached significance only for the long-range category. Differences between groups on the right side failed to reach significance (data not shown). **B** Similar effects of enrichment appeared in Sp5C where, on the left side, the distribution of maximum tree lengths increased significantly with respect to controls (*p* = 0.042; two-tailed Kolmogorov–Smirnov *D* test), as did the proportion of neurons with longer trees (*p* = 0.029), together with a decrease of those with shorter trees (*p* = 0.030). Again, no significant differences were observed on the right (data not shown)

Moreover, not all segment types were likewise affected by enrichment. In Pr5, intermediate segments of any order barely changed on the left side, but those of lower order increased in length on the right. Terminal segments, however, showed marked and significant reductions in length on the left side, an effect which failed to appear only in the highest order segments (Fig. 3). In Sp5C, terminal segments increased in length on both sides with respect to controls, although this increase reached significance only on the right side (Fig. 4). Finally, there was a tendency to a general increase in maximum Euclidean length in both Pr5 and Sp5C neurons, resulting from a relative increase in neurons with dendrites reaching longer distances, and a parallel decrease of those with shorter range dendrites. This effect reached significance only on the left side (Fig. 5).

**Fig. 6:**
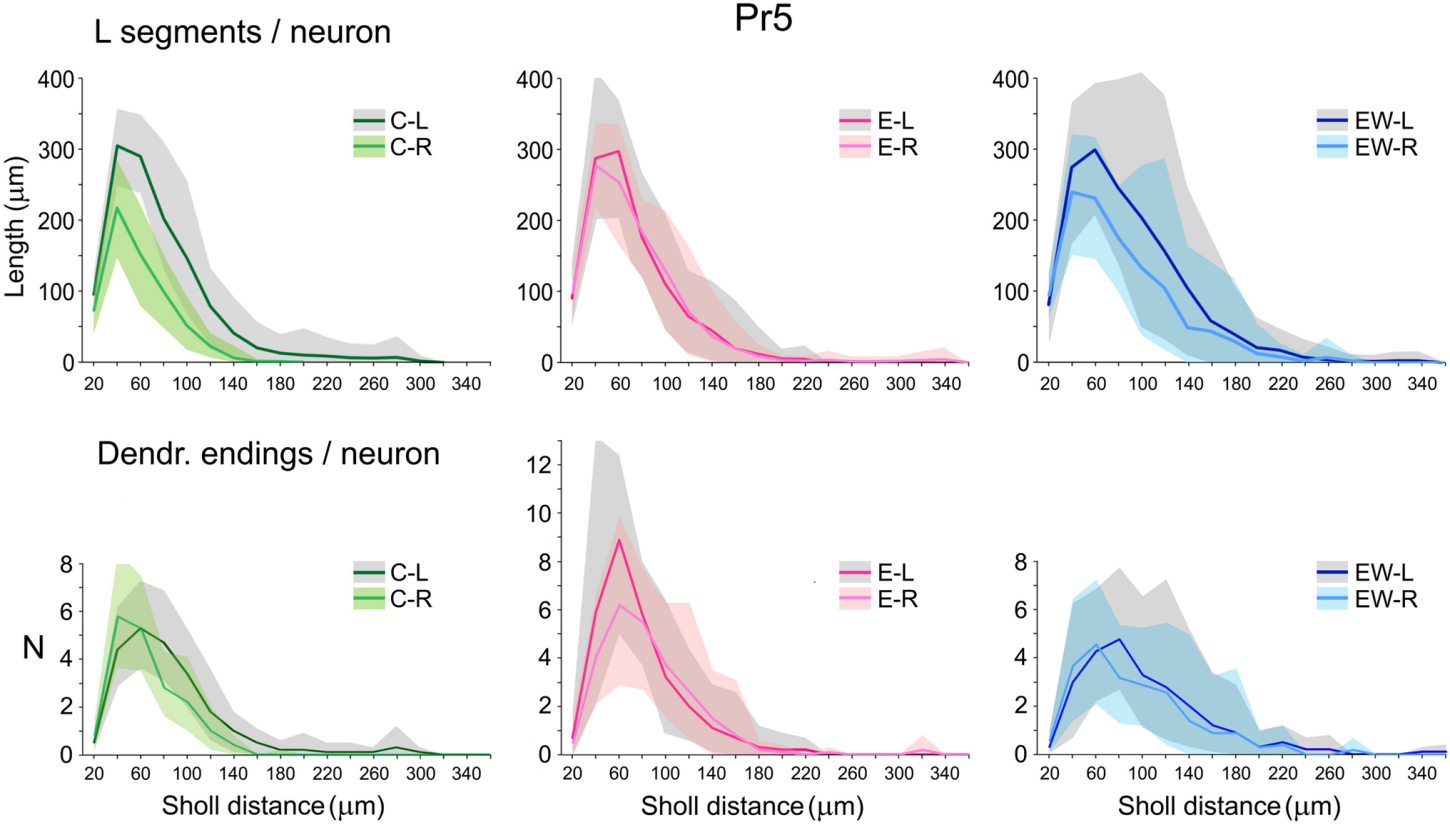
Sholl analysis showing the mean length of dendritic segments (top graphs) and number of dendritic endings (bottom graphs) per Pr5 neuron within concentric spheres from the soma center at 20 μm radial intervals. Shadings show bootstrap 90% confidence intervals (see Methods). Dark-colored lines and light-colored lines represent the left and right sides, respectively. Left, middle and right graphs correspond to the control (C), enriched (E) and enrichment-withdrawal (EW) groups. Comparisons between sides showed greater global dendritic length on the left side in controls at 40 μm from the soma and beyond, which reached significance at 60 (*p* = 0,001, two-tailed Mann–Whitney *U* test), 80 (*p* < 0.001), 100 (*p* = 0.002) and 120 μm (*p* = 0.010). Not reaching statistical significance, though, the number of dendritic endings on the left side of controls also exceeded that on the right at 80 μm and beyond

**Fig. 7:**
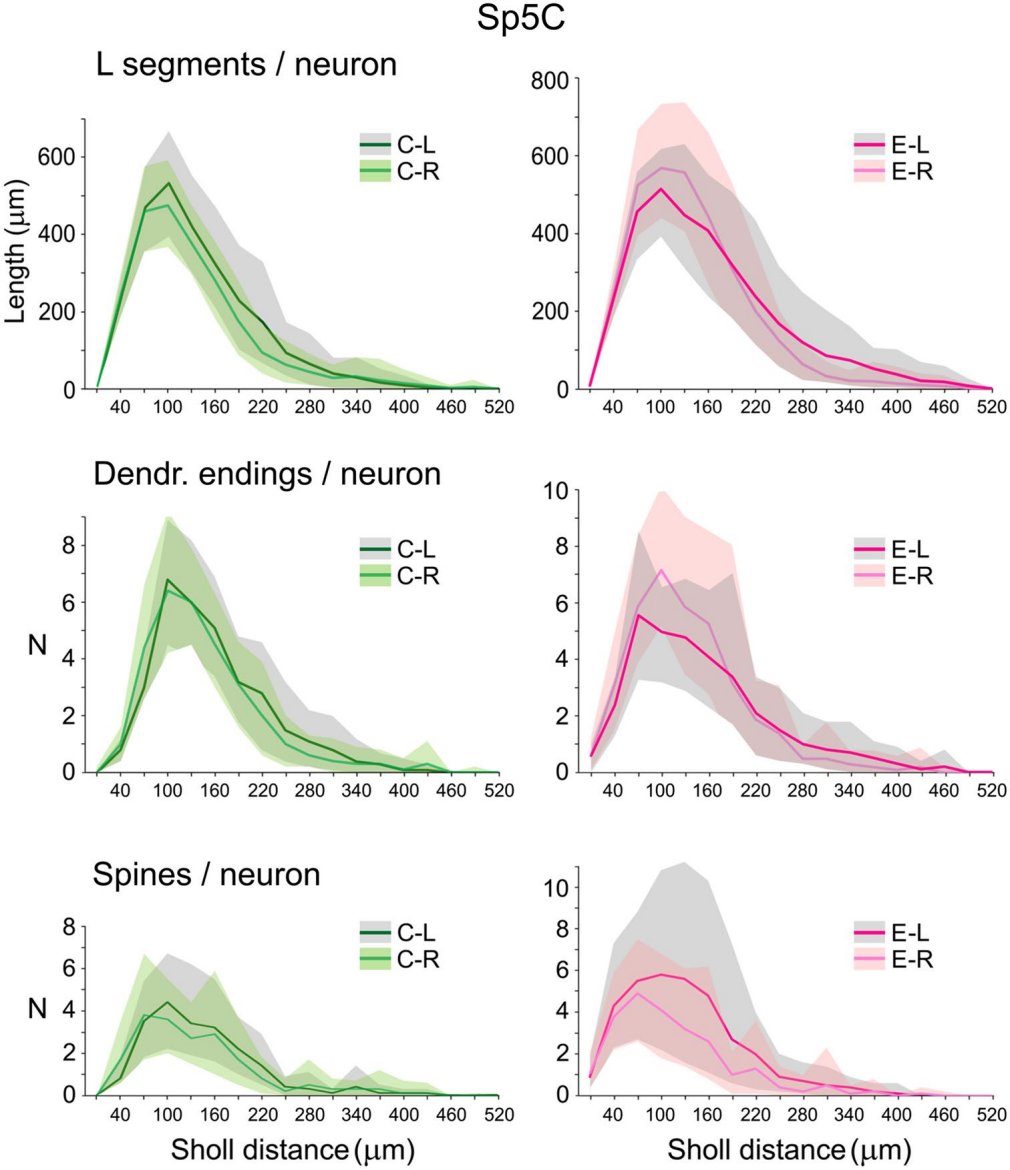
Sholl analysis showing the mean length of dendritic segments (top), number of dendritic endings (middle) and number of spines (bottom) per Sp5C neuron within concentric spheres from the soma center at 30 μm radial intervals. Confidence intervals, symbols and color codes as in Fig. 6. Comparisons between sides showed no statistically significant differences for any parameter in either group. Spines, however, showed a consistent increment on the left side after enrichment in mean total numbers per neuron (+ 45%) and densities per dendrite length (+ 32%), although not reaching significance because of a large interindividual variance

The comparisons between groups benefited from the Sholl analysis, which allowed to determine the spatial distributions of morphometric and topological parameters of the dendritic trees at fixed intervals from the neuronal somata. These intervals were set at 20 μm for Pr5 neurons and at 30 μm for the more extensive Sp5C neurons. The distribution curves of segment length and number of intersections were similar in all cases, as were the number of dendritic nodes and endings, hence only data for segment length and endings will be presented in the following Figures. The overall effect of enrichment on Pr5 neurons was the disappearance of the notable asymmetries observed in controls (Fig. 6). This effect originated from an expansion toward greater distances from the soma of the various dendritic parameters on the right, without similar changes on the left. In Sp5C neurons, a similar, but much weaker effect of enrichment on the right failed to alter the lack of asymmetries in the same parameters in controls. The number of spines, which were similar on both sides in controls, expanded notably on the left in 8/28 neurons after enrichment, an effect that did not reach statistical significance (Fig. 7).

**Fig. 8:**
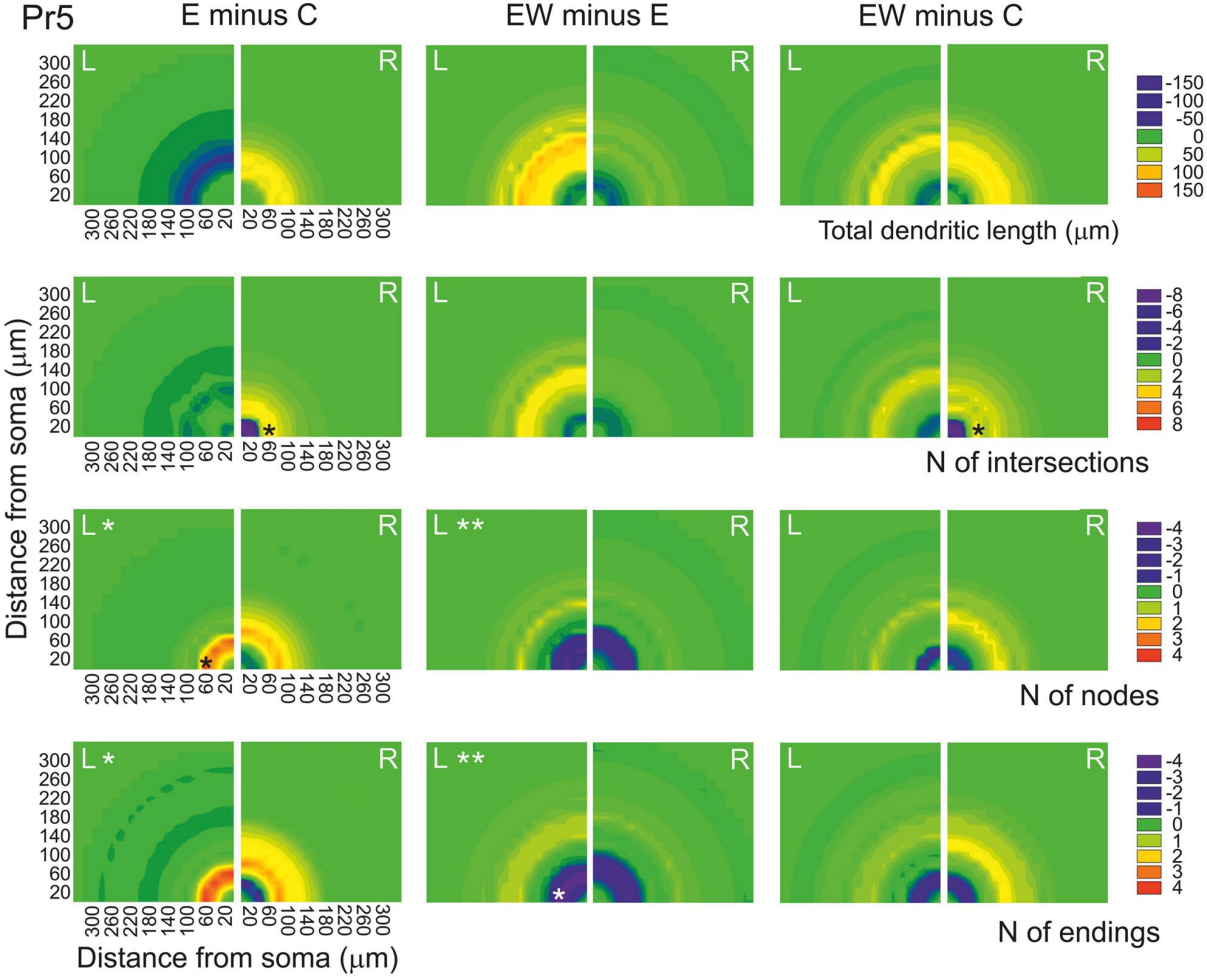
Two-dimensional color contour maps that summarize the mean changes that dendritic trees of Pr5 barrelette neurons exhibit between groups, at growing radial distance from the soma, and separated by side. The color scales were arbitrarily set between ± the average of the five largest individual values for each parameter at any Sholl distance in the groups being compared. The homogeneous distribution within a quadrant is for illustrative purposes only, and does not imply an isotropic 2D or 3D distribution of events. 3D Sholl steps = 20 μm; 2-way ANOVA with post-hoc tests. Large asterisks at the top of a quadrant indicate global statistical significance; smaller asterisks at the bottom mark the distances at which the differences reached statistical significance (*p < 0.05; **p < 0.01). Succinctly, the maps show that 1, enrichment increased the number of nodes and endings on the left, and moved intersections and endings away from the soma on the right; 2, withdrawal of the enrichment reduced the post-enrichment increase of nodes and endings on both sides, so that EW cases became more similar to controls; and 3, an exception to this pattern was the maintenance of the distancing of intersections from the soma on the right, which was similar in E and EW groups

Intergroup comparisons by side with controls are summarized in the color contour maps shown in Figs. 8 (left column) and 9. In brief, changes on the left Pr5 caused by the enrichment occurred between 40 and 120 μm from the soma, and consisted in an increase in the number of nodes and endings, combined, however, with a trend towards a decrease in total dendritic length at that interval. On the right, a tendency to greater values in all parameters at the same intervals was compounded by a reduction of intersections, nodes and endings closer to the soma (although none of these reductions reached significance). In Sp5C neurons on the right dendritic length and intersections increased significantly at 120–240 μm from the soma, with moderate increases as well in number of nodes and endings. The same occurred in the number of intersections on the left, together with a marked increase in number of spines at 60–240 μm from the soma.

**Fig. 9:**
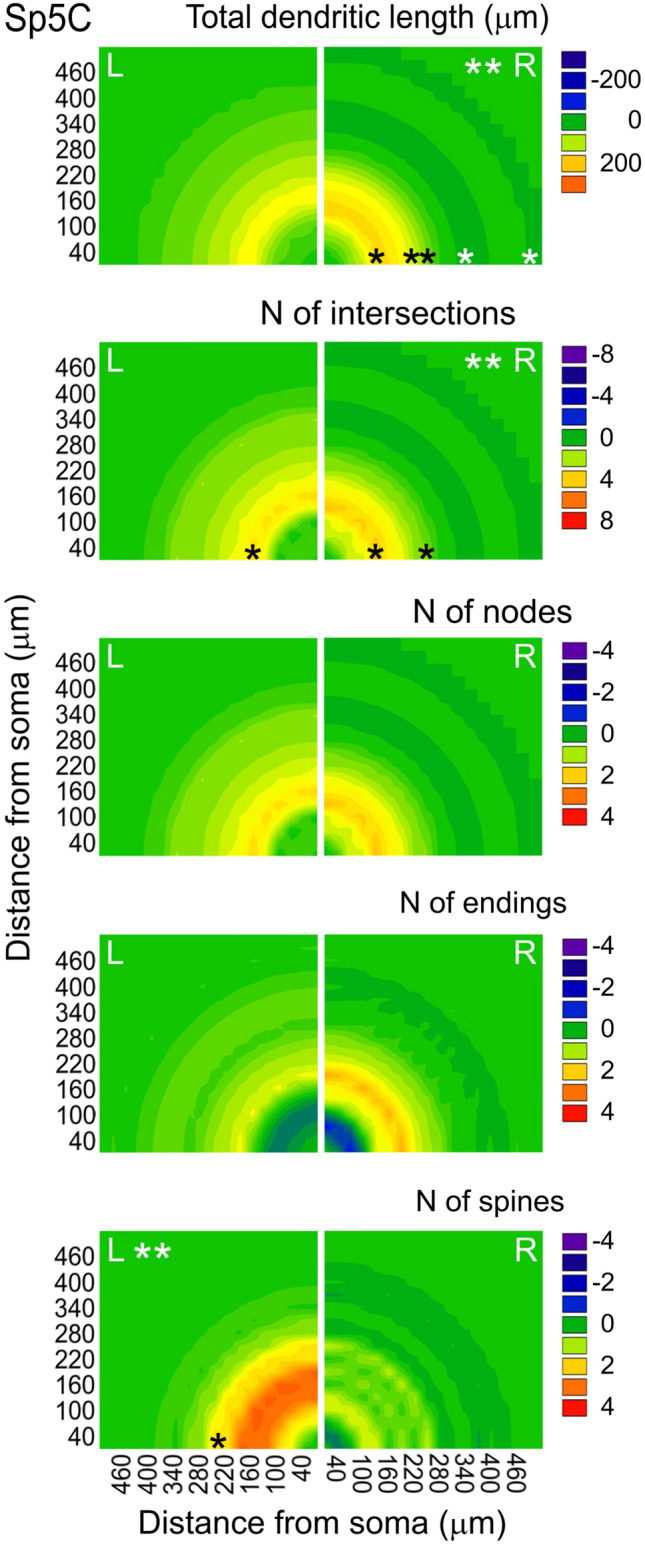
Color contour maps showing the mean changes in measures of dendritic trees of Sp5C intersubnuclear neurons after exposure to enrichment, compared to controls. Procedures, codes and criteria for this sketch as in Fig. 8, except for using here 30 μm Sholl steps. The main findings shown in the Figure are 1, a large increase in total dendritic length and number of intersections on the right side within 300 μm from the soma; 2, the increase in length turns into a decrease at longer distances from the soma; and 3, the number of spines and intersections increase between 100 and 250 μm on the left side

### Partial reversal of morphometry after withdrawal of enrichment

Two months after animals were no longer exposed to an EE, the dendritic trees of Pr5 neurons (Sp5C neurons were not studied using this paradigm) returned to values more similar to those in control cases, but not in all parameters. In particular, total dendritic length remained elevated on the right, and increased even further on the left, where dendrites reached significantly greater Euclidean distances than in C and E groups (Table 1, Fig. 5). In contrast, the number of primary dendrites, nodes, and terminal segments, which had increased after enrichment, returned to control values on the right, as did the number of more complex (high-order) dendritic trees, which had increased after enrichment on the left (Table 1 and Fig. 2). The significantly longer dendrites in the E-W group could reflect the bilateral expansion of some intermediate and terminal segments of low and medium order (Fig. 3), an expansion that occurred at distances of over 60 μm from the soma (Fig. 6). Consistent results were obtained with the Sholl analysis, which showed a disappearance of the enrichment-induced increase of nodes and endings on both sides, thus returning to values closer to controls, while the pattern of dendritic lengths and intersections across the Sholl spheres remained similar to that in the E group (Fig. 8).

## Discussion

The present study shows that discontinuous periods of exposure to a sensory-enriched environment during 2 months bring forth metric and topological changes in the dendritic trees of different populations of neurons in trigeminal nuclei of young adult male rats. Pr5 barrelette neurons, which receive primary afferents from vibrissae and project to the ventroposterior thalamic nucleus in a topologically consistent fashion (Lo et al. 1999; Veinante and Deschênes 1999; Negredo et al. 2009; Jacquin et al. 2014), show substantial side asymmetries in rats housed in standard cages (Negredo et al. 2009; present results), which disappear in rats exposed to EE. This effect mainly results from an increase, only on the right side, of global dendritic length, number of trees, nodes and terminal segments, and length of intermediate segments. On the left side, trees shrank in length at 40–120 mm from the soma, raising the number of nodes and endings in that interval. Sp5C neurons also receive innervation from primary trigeminal afferents, and send focused intersubnuclear projections to the barrelettes of Pr5, which play a significant role in shaping the receptive fields of thalamic-projecting Pr5 neurons (Timofeeva et al. 2004; Furuta et al. 2008; Jacquin et al. 2014; Martin et al. 2014). Changes in these neurons after enrichment were more limited, mainly consisting of a significant global increase in dendritic length on the right, concentrated at intermediate distances from the soma where more numerous intersections, nodes and endings appeared. On the left side, dendrites reached greater maximum Euclidean distances from the soma, and displayed more dendritic spines. To assess the permanence of the effects of enrichment, the dendritic trees of Pr5 neurons were analyzed when animals exposed for 2 months to an EE were returned for another 2 months to the same standard housing as our controls. Some parameters proved temporary, reverting to control values, except for the total dendritic length, which remained significantly greater on the right and increased even more on the left, reaching greater maximum Euclidean distances.

Exposing laboratory animals to more complex environments than is customary under standard housing conditions has patent effects on brain structure and function and behavioral abilities. While the younger the animals, the more varied and prominent these effects are, some can be elicited in adult and even aging subjects. This form of experience-dependent plasticity is more salient in the cortex, which, unsurprisingly, has attracted most attention from researchers. Adult rodents exposed to variable periods of different types of EE showed global changes in the cortex, such as an increment in weight and thickness (Rosenzweig et al. 1962; Machín et al. 2004), increased length, branching of dendrites and dendritic spines, although only in some cortical regions and sectors of the dendritic arbors (Volkmar and Greenough 1972; Greenough et al. 1973; Globus et al. 1973; Connor et al. 1981, 1982; Kolb et al. 2003; Gelfo et al. 2009; Bose et al. 2010), and greater synaptic density (Turner and Greenough 1985; Landers et al. 2011). These structural changes are accompanied by significant enhancements of functional aspects of cortical sensory processing (Frostig 2006; Devonshire et al. 2010; Kalogeraki et al. 2017; LeMessurier et al. 2019).

Comparatively, the effects of enrichment on brain or spinal structures outside the cortex have been largely neglected. Exceptions exist, however, such as the observation of increased dendritic arborization and spines in nucleus accumbens (Kolb et al. 2003), the increment in thalamus and hypothalamus of biochemically detected levels of two synaptic proteins, synaptophysin and PSD-95 (Nithianantharajah et al. 2004), or an increased sprouting of incoming retinal axons and recovery of synaptic molecular markers in the partially deafferented superior colliculus (Caleo et al. 2009). More remarkably, the primary sensory neurons themselves reveal molecular and structural modification in adult rats following long-term exposure to EE: Genes coding for some glutamate receptor subunits and proteins involved in clustering and stabilization of AMPA receptors in the trigeminal ganglion display left–right asymmetries that grow after enrichment (Fernandez-Montoya et al. 2016). Moreover, enrichment resulted in significant increases in the number and size of varicosities of primary afferent myelinated fibers from vibrissal follicles in all trigeminal nuclei (Fernández-Montoya et al. 2018).

Little attention has been paid to the duration of the neural changes upon withdrawal of the exposure to enriched environment. Behavioral studies showed lasting persistence of a number of measures of emotional reactivity, exploratory behavior and enhanced learning brought about by enrichment, 1–3 months after withdrawal from the enriched environment (Briones et al. 2004; Peña et al. 2009; Zocher et al. 2020). In addition, increases in synapse density in visual cortex evoked by enrichment persisted 1 month after EE removal (Briones et al. 2004), and behavioral and epigenetic changes evoked by enrichment were largely maintained 3 months after withdrawal (Zocher et al. 2020). On the other hand, the lifelong persistence of ocular dominance plasticity generated in mice raised in EE disappeared 1 week after returning the animal to standard housing, although such reversion could be in turn brought back to renewed plasticity if animals were supplied running wheels in their cages to enhance voluntary physical exercise (Kalogeraki et al. 2017). To our knowledge, dendritic changes ensuing similar alternations in housing environments have not been reported. The present finding of a partial persistence of the dendritic changes induced by enrichment in Pr5, 2 months after returning to standard housing reflects the dynamic character of experience-dependent shaping of neural structures.

The asymmetry of dendritic arbors of barrelette neurons in Pr5 of naïve rats, with shorter and less extensive dendrites on the right (Negredo et al. 2009), could underpin the generation of more focused receptive fields on the right. This would be reinforced by the presence of denser and perhaps more spatially restricted terminal bushes of primary afferents on the right Pr5 (Fernández-Montoya et al. 2018). Moreover, the loss of haptic touch from the right whisker pad brought about reductions in the spatial extent, but increased branching of Pr5 barrelette neurons’ dendrites on the left, lending support to the idea that these structural changes occur in response to the side transfer of somatosensory-guided tasks after unilateral deprivation (Negredo et al. 2009). In the present study, enrichment brought about a significant increase in total dendritic length on the right Pr5, but this occurred by an increase in dendritic branches at close quarters with the cell body, without overall spatial expansion as indicated by the Sholl analysis. Increased branching was observed bilaterally, as indicated by higher numbers of nodes and terminal segments on both sides. Together with the increased number and size of terminal and *en passant* varicosities in primary afferents ending on the (right) Pr5 (Fernández-Montoya et al. 2018), our findings suggest that an enhanced active exposure to sensory input could impose the growth of further dendritic domains, without altering much the overall spatial reach of barrelette neurons’ dendritic trees. These results in lower stations of the trigeminal pathways are accompanied by similar plastic changes in the barrel cortex after enrichment, which include, among other features, growth in length of dendrites and formation of new spines and synapses (Connor et al. 1982; Landers et al. 2011). Interestingly, such structural expansions, however, come with smaller and sharper receptive fields and finer whisker tuning in neurons of layers II–IV (Frostig 2006; LeMessurier et al. 2019).

In sum, this study advances the notion that sensory experience-dependent plasticity is a ubiquitous phenomenon across the neural systems involved in sensory processing. By solely focusing on the, admittedly more conspicuous, cortical plasticity, we may inadvertently overlook critical sensory input-dependent changes that take place at early stages of sensory processing. This ‘more peripheral’ plasticity should be tackled to better understand the processes of learning and adaptation associated with exposure to changing and more complex environments. And more so when this type of exposure is in recent years moving past the laboratory into clinical settings as an ancillary tool in the treatment of, or rehabilitation from a number of brain pathologies or neuropathic pain (Nithianantharajah and Hannan 2006; McDonald et al. 2018; Kimura et al. 2020). This, in addition, highlights the need to explore sensory experience-dependent structural dendritic plasticity in subjects of both sexes, since sex-dependent variations in dendritic plasticity have been reported in hippocampus and other higher centers under different conditions (Hyer et al. 2018).

## Supplementary Information

Below is the link to the electronic supplementary material.

Supplementary file1 (PDF 247 KB)

## Data Availability

The datasets generated for this study are available on request to the corresponding author.
