# Supplementary material for: Experience-dependent plasticity in early stations of sensory processing in mature brains: effects of environmental enrichment on dendrite measures in trigeminal nuclei
Source: Brain Struct Funct. 2021 Nov 22;227(3):865–79. doi: 10.1007/s00429-021-02424-3 (PMC8930882; doi:10.1007/s00429-021-02424-3)

Supplementary Fig. 1 Photomicrographs to illustrate tracer injections, retrogradely labeled neurons, and details of dendrites in cases injected in VPM (top row, A-E) and Pr5 (bottom row, F-J). A and F show DAB-reacted BDA thalamic and trigeminal nucleus injections, respectively. B and G show adjacent cytochrome oxidase-reacted sections to facilitate nuclear delineation. C and D represent examples of trigeminothalamic *barrelette* neurons from the left (C) and right hemisphere (D) of a control and an enriched case, respectively. The dendrites of these cells are esentially spine-free (E). H and I show examples of intersubnuclear Sp5C neurons from the right hemispheres of a control (H) and an enriched (I) case. These neurons are consistently sparsely populated with spines (some marked with arrows, J). *Scale bars*, 500 μm (A,B), 200 μm (F,G), 20 μm (C,D,H,I), and 10 μm (E,J).

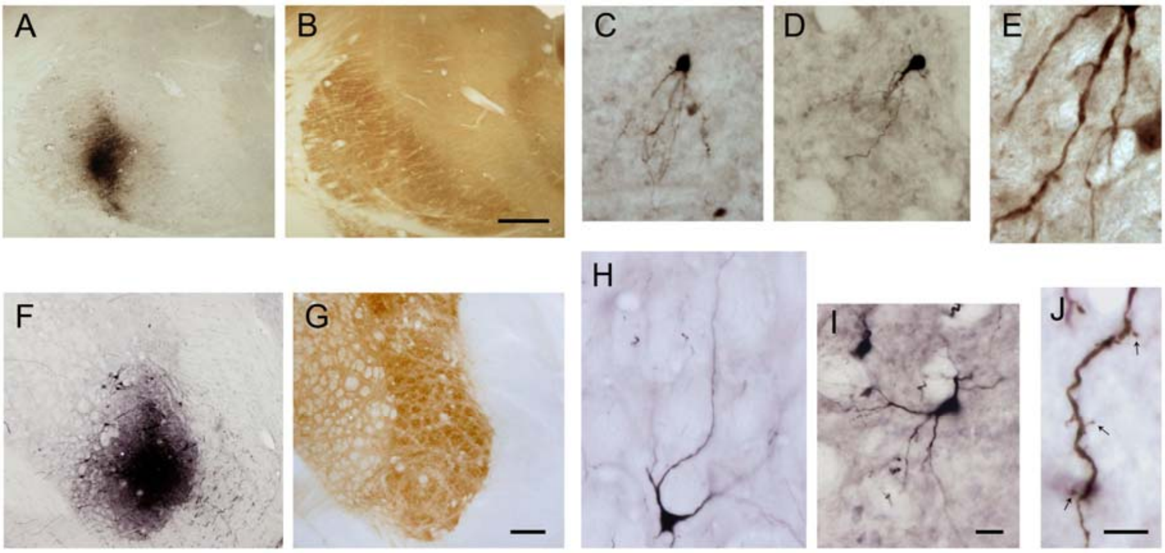

Supplementary Fig. 2 Examples of digital reconstructions in the coronal plane of trigeminothalamic *barrelette* neurons in Pr5 (top) and intersubnuclear Sp5C neurons (bottom). The initial portion of their axons is indicated with small arrows. Cells in control Pr5 correspond to representative barrelette neurons, in terms of total dendritic length, in the mystacial vibrissae-receptive zone of Pr5 (highlighted in gray in the insets). The neuron reconstructed from the right side of an enriched case has a total dendritic length similar to that shown from the control left side shown here (although it may appear larger on a single plane projection). Neurons in the Sp5C are often located in laminae II (highlighted in gray) and III. Those reconstructed from control cases represent neurons of an average size, while those from enriched cases correspond to examples of neurons with the largest dendritic arbors, which are never found in controls. *Scale bar* 100 μm.

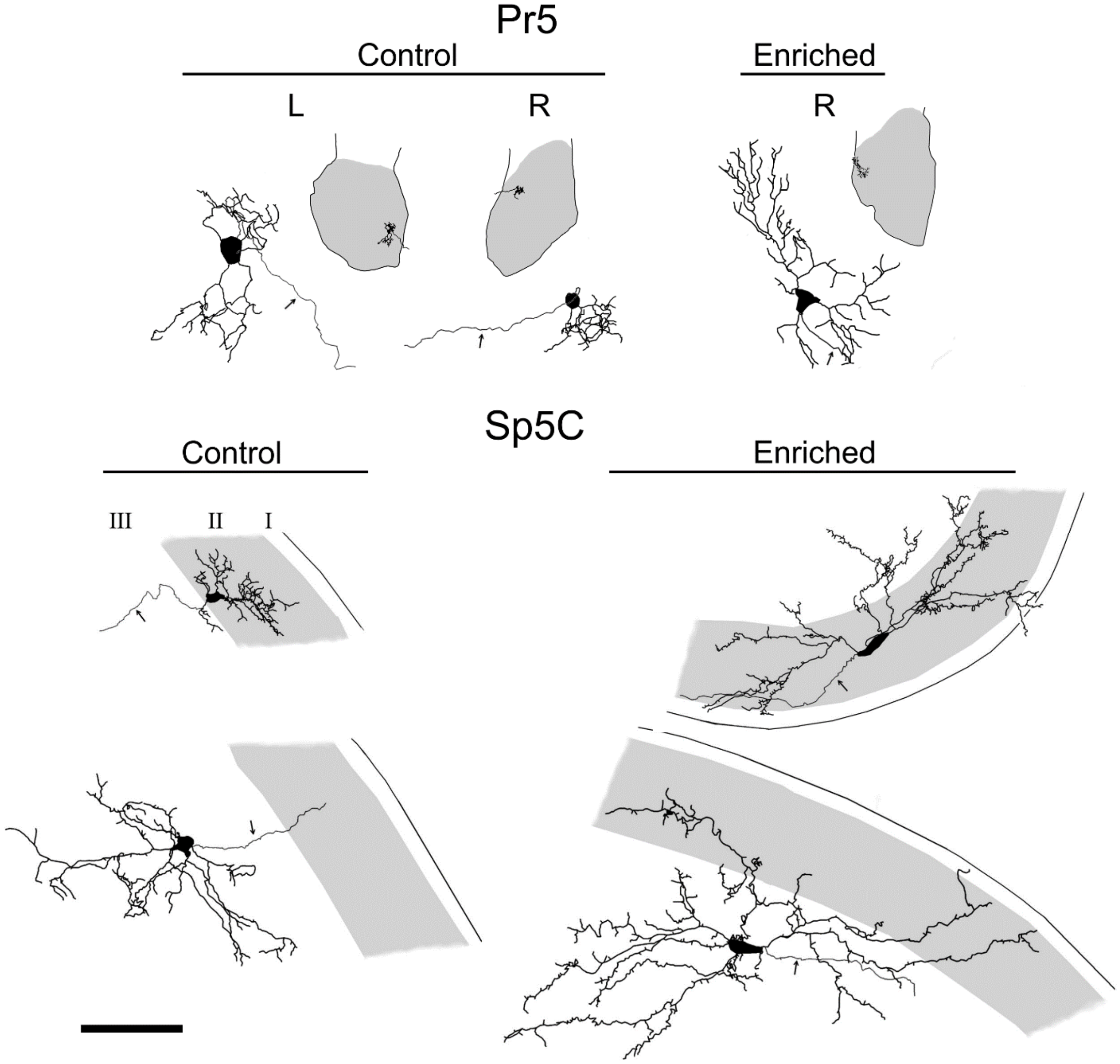

Supplement: Supplementary file 1 — Supplementary file1 (PDF 247 KB) [file 429_2021_2424_MOESM1_ESM.pdf]
